# The effects of oxygen flow ratio on the properties of Ag_*x*_O thin films grown by radio frequency magnetron sputtering †

**DOI:** 10.1039/d4ra02039a

**Published:** 2024-07-23

**Authors:** Xiaojiao Liu, Tatsuya Yasuoka, Giang T. Dang, Li Liu, Toshiyuki Kawaharamura

**Affiliations:** a Engineering Course, Graduate School of Engineering, Kochi University of Technology 185 Miyanokuchi, Tosayamada, Kami Kochi 782-8502 Japan 248009y@gs.kochi-tech.ac.jp kawaharamura.toshiyuki@kochi-tech.ac.jp +81-887-57-2703; b School of Systems Engineering, Kochi University of Technology 185 Miyanokuchi, Tosayamada, Kami Kochi 782-8502 Japan; c Center for Nanotechnology, Research Institute, Kochi University of Technology 185 Miyanokuchi, Tosayamada, Kami Kochi 782-8502 Japan

## Abstract

The Ag_*x*_O thin film with various oxygen flow ratios (*R*[O_2_]%) deposited by radio frequency magnetron sputtering (RFM-SPT) has been studied. While adjusting *R*[O_2_]% from 0% to 30%, the Ag_*x*_O thin film transitioned from metal to semiconductor and/or insulator with different transparent appearances on the surface observed using X-ray diffraction (XRD) and transmittance measurement. At high oxygen flow ratios, the Ag_*x*_O film is multi-phased as a mixture of Ag^(II)^O and Ag_2_^(III)^O_3_. In addition, the work function (*ϕ*) of those samples changes from 4.7 eV to 5.6 eV as measured by photoelectron yield spectroscopy (PYS). The compositional and chemical state changes that occur at the Ag_*x*_O surface during the increments of *R*[O_2_]% are evaluated by the relative peak intensities and binding energy shifts in X-ray photoelectron spectroscopy (XPS). With the incorporation of more electrons in chemical bonding, the oxygen-induced band forms. And combining all the results from transmittance (band gap confirmation), PYS (work function confirmation), and XPS (valence band position confirmation), the estimated band diagrams are given for the oxidation state of Ag_*x*_O with various oxygen flow ratios.

## Abstract 2

When silver oxide was deposited using sputtering, its color changed depending on the oxygen supplied amount. How would the oxygen flow ratio affect the properties of Ag*_x_*O thin films prepared by radio frequency magnetron sputtering?

## Graphical abstract

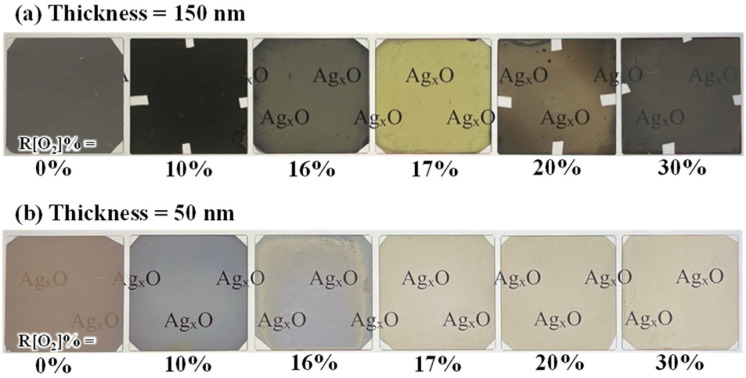

## 1. Introduction

Oxide-based thin film properties are highly beneficial in various technological applications including transparent conductive films, gas sensors, LEDs, transparent electronic devices, and solar cells, among others. Thus, to obtain the adjustable properties of semiconductor materials, precise control of the extent of oxidation of thin film during the synthesis process is important for the electronics industry.^1–3^

According to previous investigations, silver oxide (Ag_*x*_O) is a transparent material in wavelengths ranging from the infrared region to the visible region with an optical band gap in the range of 1.2–3.1 eV. Whereas, silver (Ag) is a good reflective coating for applications in the optoelectrical field,^4^ Ag_*x*_O is preferable for use in battery electrodes as it performs better in voltage regulation and enhances storage life.^5,6^ The work function can reach roughly 5.3 eV with O_2_ plasma or UV-ozone treatment of Ag anodes.^7,8^ In particular, Ag_2_O films in general are reported to show p-type semiconducting properties.^9–11^ Ag_*x*_O has attracted attention as an air electrode in fuel cells and metal–air batteries because of its relatively low Ag–O bonding energy (221 kJ mol^−1^)^12–17^ enabling it to interact with oxygen reactants and their intermediates without excessively covering the catalyst surface. However, previous studies^7,18^ have revealed that the instability of Ag_*x*_O resulting from heating is in the transition of its composition, crystallinity, refractive index, and other properties. Furthermore, silver oxides (AgO, Ag_2_O) desorb oxygen at low temperatures (∼200 °C),^18^ in consequence, a low-temperature film fabrication process is required to form Ag_*x*_O thin films.

Sophisticated growth methods for Ag or Ag_*x*_O thin film depositions are well developed, including chemical bath deposition (CBD),^10^ dry chemical route at room temperature,^11^ electron beam (EB) evaporation,^18,19^ microwave sputtering,^20^ and DC magnetron sputtering.^21–24^ In addition, partially oxidized Ag_*x*_O films grown *via* reactive sputtering were applied for the fabrication of Ag_*x*_O/ZnO Schottky diodes (SBDs),^25^ Ag_*x*_O/α-Ga_2_O_3_ metal semiconductor field effect transistor (MESFET),^26^ Ag_*x*_O/IGZO SBDs,^27^ and Ag_*x*_O/ZnMgO Heterojunction diodes (HJDs).^28^ Those applications of Ag_*x*_O thin films were proven to improve the barrier height in the interface region of the heterojunction. Indeed, the reactive oxygen might have the capacity to provide O-rich ambient healing of the interface defects or states, for during the sputtering deposition process Ag ion/plasma would combine with reactive oxygen (oxygen plasma, oxygen radicals). Hence, the purpose of this work is to investigate different oxygen flow ratios of *R*[O_2_]% = [O_2_/(O_2_ + Ar)]% effects on the Ag_*x*_O film properties grown by the radio frequency magnetron sputtering (RFM-SPT) deposition method.

## 2. Experimental methods

Ag and Ag_*x*_O films were deposited at room temperature (RT) on quartz substrates which were placed vertically above the Ag target (99.99% purity, 3 inches in diameter) at an appropriate distance of 20 cm by introducing the excited gas mixture of Ar without/with O_2_*via* a radio frequency magnetron sputtering (RFM-SPT) deposition system. Prior to the film deposition, the reaction chamber was evacuated to a base pressure below 1 × 10^−4^ Pa. During film deposition, working pressure was maintained at 0.3 Pa. The total flow rate ([O_2_ + Ar]) was set at 10 sccm, and the oxygen flow ratio (*R*[O_2_]% = [O_2_/(O_2_ + Ar)]%) was changed from 0% to 30% while the power supply was fixed at 40 W, and the reflected power was controlled in the range of 0.5–1 W during the deposition process. Each quartz substrate was ultrasonically precleaned for 2 min in acetone, isopropyl alcohol, and deionized (D. I.) water, respectively, and was then dried using an N_2_ gas gun. Before checking the growth rate, the pretest sample preparation was operated for maintaining the chamber ambient and also for removing the top oxidized layer of the Ag target, after which the thin film with target thickness was prepared accordingly. Details of the deposition conditions are summarized in Table 1.

**Table 1:** RFM-SPT deposition conditions for Ag_*x*_O films

Target	*R*[O_2_] (%)	Pressure (Pa)	Power (W)	Thickness (nm)	Growth time (min)
Ag	0–30	∼0.3	40	50	≅5
				150	≅20

The crystal structure was analyzed by grazing incident X-ray diffraction (GIXD) spectra using CuKα radiation (Rigaku Corp., Smart Lab, X-ray wavelength *λ* = 1.5418 Å, incident angle 0.35°). When specifying the material, information on each crystal was obtained from the works of literature,^29–34^ the angle at which the specific X-ray diffraction of Cu was determined, and the measurement results were compared with the theoretical diffraction angle, identifying the material. The thickness of the obtained coatings was measured by a step profiler in advance of the growth rate checking, and after that, the formal deposition with target thickness was operated. The surface morphology was characterized by scanning electron microscopy (SEM) (Hitachi SU8020) with an acceleration voltage of 5 to 15 keV. The optical transmittance of Ag and Ag_*x*_O films was measured in the range from 200 nm to 2500 nm using a UV-visible spectrometer (Hitachi UV-vis U-4100). Afterward, the band gaps were calculated and confirmed by the Tauc plot. The Tauc plot was built on the data extracted from the transformation of transmittance spectra into absorption spectra. The resistances were measured by the resistivity measuring system (MITSUBISHI Chemical Corp., Hiresta-Up MCP-HT450). The work function of 150 nm thick Ag and Ag_*x*_O films on the quartz substrate was determined by photoelectron yield spectroscopy (PYS) (BUNKOUKEIKI Corp., BIP-KV202GD/UVT) in a vacuum at room temperature. X-ray photoelectron spectroscopy (XPS) (Ulvac-Phi ESCA 5500 A) was used to investigate the chemical states of Ag and Ag_*x*_O films, and a monochromic Al Kα (1486.6 eV) X-ray source was operated at 96 W and 12 kV at room temperature below 3.0 × 10^−8^ Pa. The binding energies in the XPS spectra were calibrated by the peak position of C 1s spectra appearing at 284.8 eV for adventitious carbon surface contamination.^35^

## 3. Results and discussion

**Fig. 1:**
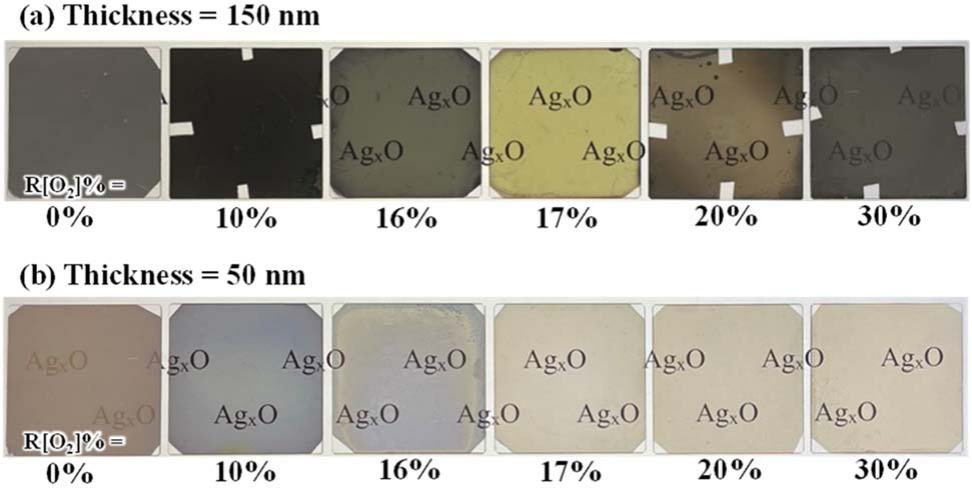
Ag_*x*_O samples appearance photos comparison with various *R*[O_2_]% (a) thickness is 150 nm (b) thickness is 50 nm.

Under vacuum ambient, Ag_*x*_O films were deposited on quartz in various oxygen flow ratios (*R*[O_2_]%). Photos showing the appearance of Ag_*x*_O, which had lengthy exposure to the atmosphere and was put on top of the illuminated screen, are shown in Fig. 1 (a) 150 nm and (b) 50 nm. At the same light illumination, Ag_*x*_O samples present different transparency colors, for the increment of *R*[O_2_]%. The color-changing tendency is the same for 150 nm and 50 nm films. More remarkable changes could be seen in (a) 150 nm samples; as Ag_*x*_O film with *R*[O_2_]% = 0% presented a silvery metallic color and 10% showed a brown dark color blocking light from passing through. But after increasing *R*[O_2_]% from 16% to 30%, all the Ag_*x*_O films showed lighter brown-grey colors except for *R*[O_2_]% = 17% which presented a yellow color. It can be seen that the appearance changes significantly in relation to the flow ratio of oxygen (the change of *x* value), and the color-changing tendency follows in 4 categories of oxygen flow ratio: 0%, 10%, below 17%, and above 17%.

**Fig. 2:**
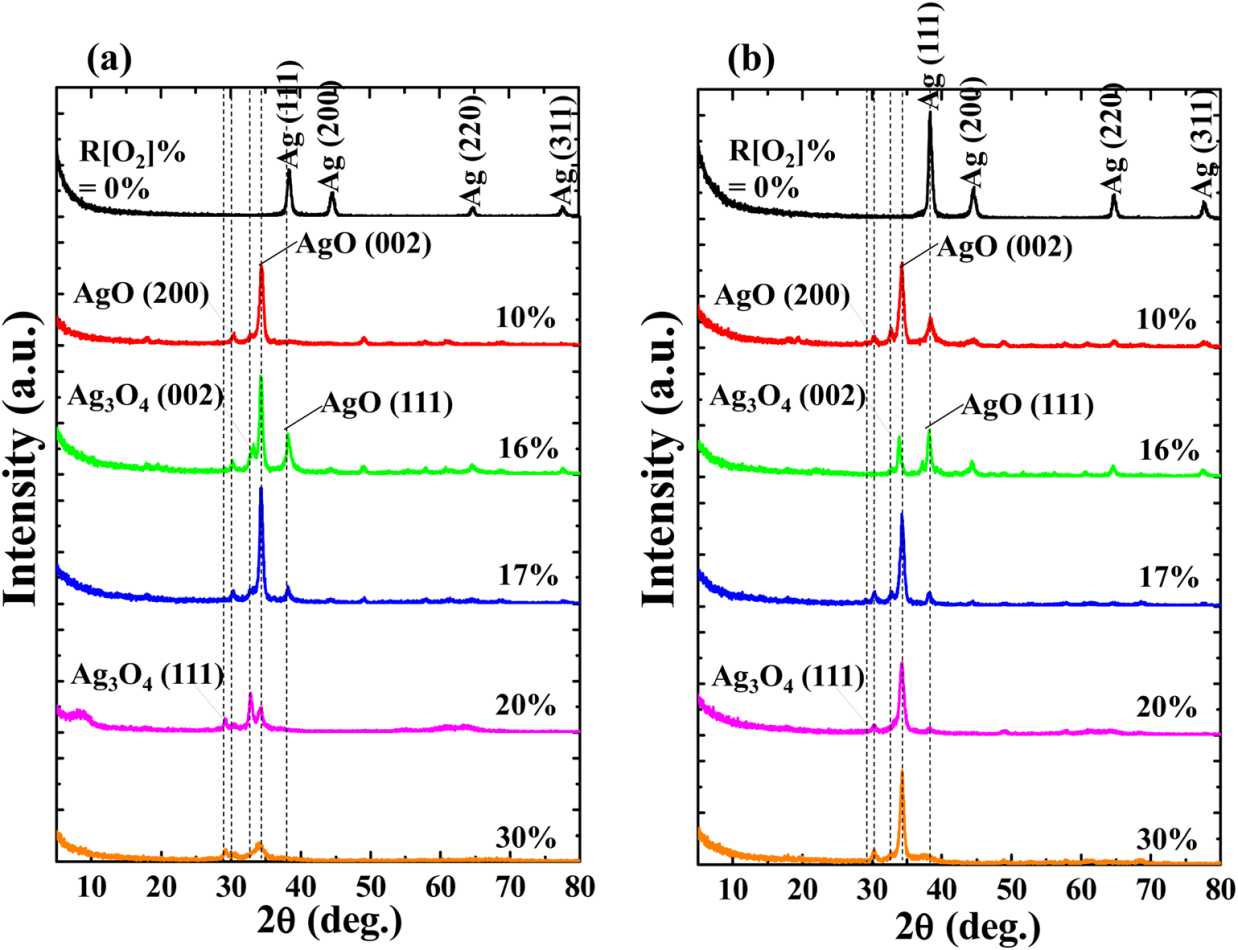
Influence of *R*[O_2_]% on GIXD of Ag_*x*_O films (a) 150 nm (b) 50 nm.

The influences of *R*[O_2_]% on crystal structure were investigated *via* GIXD spectra with the thickness of (a) 150 nm and (b) 50 nm as shown in Fig. 2. Focusing on the samples of *R*[O_2_]% = 0%, the Ag-related diffraction peaks are detected as: (111), (200), (220), and (311), respectively in both 150 nm and 50 nm thickness samples. The relative intensity of the Ag (111) diffraction peak is significantly higher than others. After introducing oxygen into the depositions, all the diffraction peaks of Ag were diminished. Ag cations started to bond with oxygen ions forming transparent Ag_*x*_O films. For the low oxygen flow ratios of *R*[O_2_]% = 10% to 17%, the dominated diffraction peak at 2*θ* = 34.52° was confirmed as the representation of AgO (002) peak,^36,37^ and its intensity is properly proportional to the increment of *R*[O_2_]%. However, when *R*[O_2_]% exceeded 20%, the intensities of AgO (002) were reduced. On the other hand, the other two related AgO peaks of (200) and (111) were detected at 2*θ* = 30.2° and 38.3° from these measured samples. AgO (200) had a relatively weak signal with constant intensity, and the AgO (111) only presented as *R*[O_2_]% = 16% and 17% with decreased intensity. No Ag_2_O peaks were detected in these depositions.^38–40^ At the same time, another small shoulder-shaped diffraction peak adjacent to AgO (002) diffraction peak was determined as the Ag_3_O_4_ (002) diffraction peak^41,42^ without regular tendency of intensities of increment of *R*[O_2_]%. After *R*[O_2_]% increased to 20% and 30%, another related to the Ag_3_O_4_ diffraction peak (111) was presented at 2*θ* = 29° with weak constant intensity. Other studies^43–45^ have reported that the dissociation of Ag_3_O_4_ (2Ag_3_O_4(s)_ → 6AgO_(s)_ + O_2(g)_) readily happen even at room temperature, and Ag_3_O_4_ crystal grains end up as randomly distributed between the Ag^(II)^O and Ag_2_^(III)^O_3_. Thus at high *R*[O_2_]%, the Ag_*x*_O grains are considered as a mixture of two or more individual oxides of Ag^(II)^O and Ag_2_^(III)^O_3_.^37,46–48^

In addition, comparing the samples with different thicknesses prepared at the same *R*[O_2_]%, it was found that the confirmed diffraction peaks appeared at the same 2*θ* position with slightly different intensities, but Ag_3_O_4_ (111) peak is not mainly presented in the 50 nm sample, and AgO (002) peak appeared as the dominant peak at any oxygen flow ratios.

**Fig. 3:**
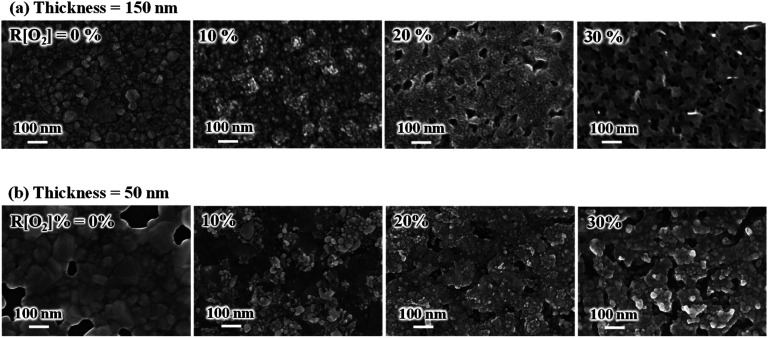
Influence of *R*[O_2_]% on the surface morphology of Ag_*x*_O films (a) 150 nm (b) 50 nm.

The surface morphology images of Ag_*x*_O (a) 150 nm and (b) 50 nm are shown in Fig. 3. Without oxygen, the Ag films of both the 150 nm and 50 nm thickness samples showed good smooth top surfaces. After introducing oxygen, the grain shape presented a spherical grain morphology and the grain size became smaller, and the white color particles were characterized by a spherical grain morphology on top of the surface. In the meantime, we can see that the Ag_*x*_O films become much looser and polyporous as the increment of *R*[O_2_]% from 20% to 30%, indicating that the surface relocation of silver atoms had increased and the 3-dimensional crystal growth formed at high oxygen flow ratios.

**Fig. 4:**
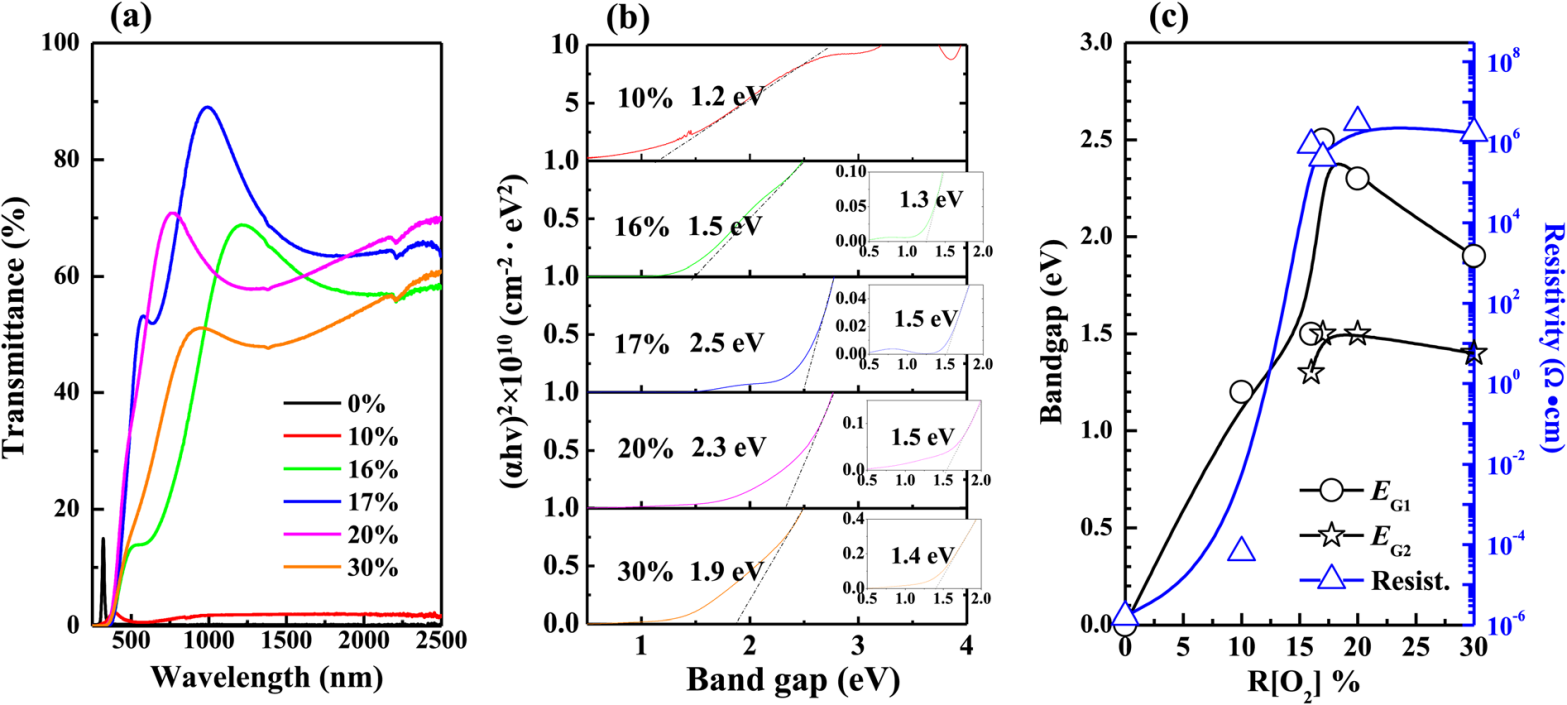
Variations in (a) transmittance (b) band gaps were extracted from the linear extrapolations of thickness 150 nm samples (c) band gaps and resistivity as a function of *R*[O_2_]%.

The variations in transmittance with the wavelength of different *R*[O_2_]% of 150 nm Ag_*x*_O films are shown in Fig. 4(a). It can be seen that the transmittances between the *R*[O_2_]% of 10% and 16% increased in the longer wavelengths. The absorption edges were narrowly blue-shifted between *R*[O_2_]% of 10–17%, but after *R*[O_2_]% increased to 20% and 30% the transmittances were decent and the absorption edges were red-shifted in a certain range. The relation between the optical absorption coefficient *α* of a direct band gap (*E*_G_) semiconductor near the band edge and the photon energy *hv* is given by the following eqn (1):^49,50^

1 *αhv* = *A*(*hv* − *E*_G_)^1/2^

where *A* is a constant value. The film transmittance (*T*) around the absorption edge is approximated as exp(−*αt*), where *t* is the film thickness. By then plotting (*αhv*)^2^*versus* photon energy *hv*, the band gap *E*_G_ can be confirmed by linear extrapolations of absorption region. In addition, referring to the data from GIXD results, knew that 150 nm samples had present mixture crystals in certain conditions. Shown in Fig. 4(b) is the band gap evaluation of 150 nm thickness Ag_*x*_O samples. The optical band gaps (*E*_G1_) widened from 1.2 to 2.5 eV as *R*[O_2_]% increased from 10% to 17%. After the *R*[O_2_]% increased to 20% and 30%, the band gap decreased. Meanwhile, the observation from Fig. 4(b) of the absorption edges curve along with different trends indicates that the Ag_*x*_O grains were a mixture of two individual oxides. When magnifying into the smaller range of absorption region as shown in the inset of Fig. 4(b), the other band gaps (*E*_G2_) value could be confirmed. And the resistivity (*ρ*) was also evaluated which had a similar changing tendency to that of band gaps confirmed variations with the increment of oxygen flow ratios, that is as *R*[O_2_]% = 10–16% *ρ* increased from 6.3 × 10^−5^ Ω·cm to 8.6 × 10^5^ Ω·cm and as *R*[O_2_]% = 17–30% *ρ* slightly decreased but almost stabilized as 10^6^ Ω·cm. Whereas the thickness of the films affected the optical evaluation, we decided to evaluate thinner samples with the thickness of 50 nm again.

**Fig. 5:**
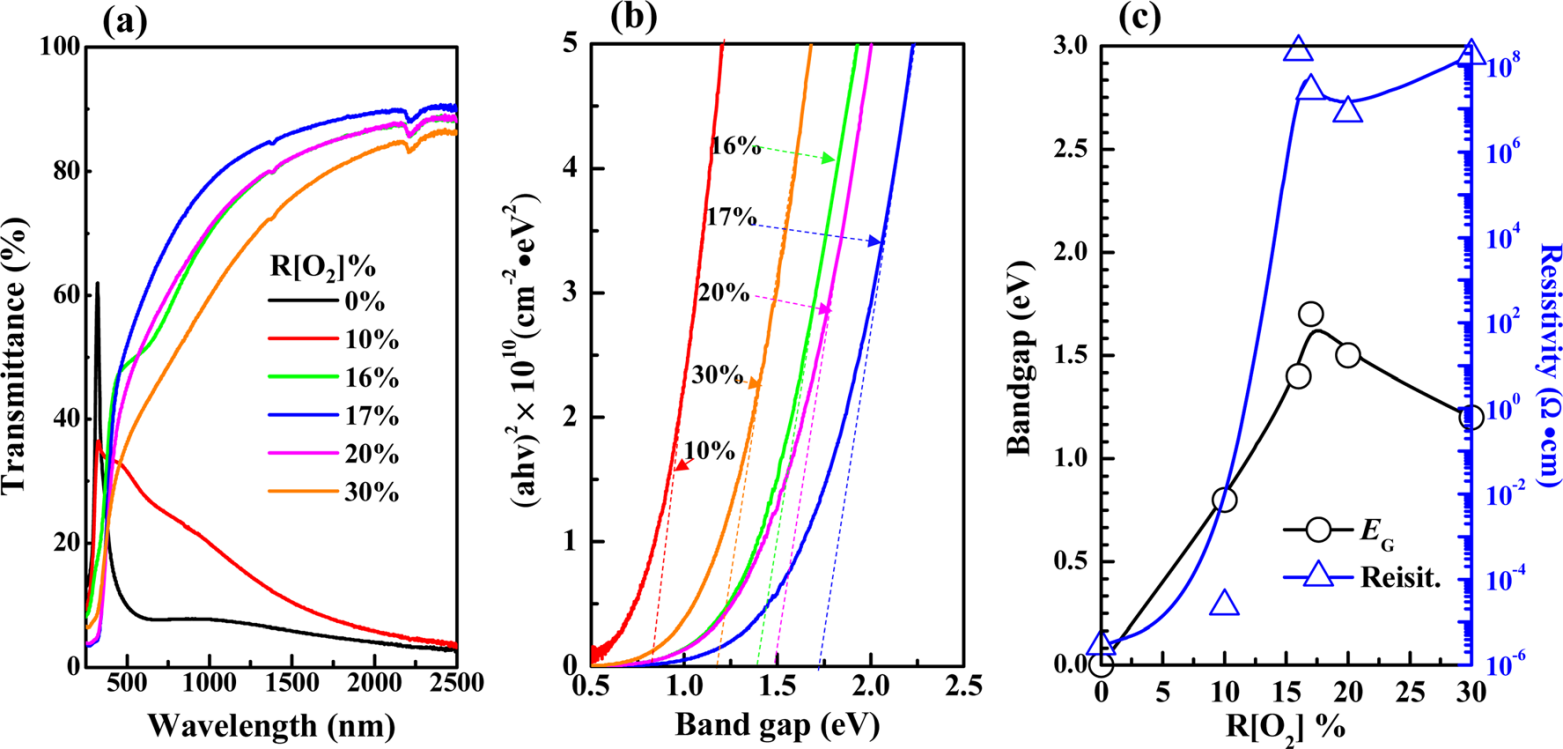
Variations in (a) transmittance (b) band gaps were extracted from the linear extrapolations of thickness 50 nm samples (c) band gaps and resistivity with different *R*[O_2_]%.

Fig. 5 shows the optical properties of 50 nm Ag_*x*_O films, which were more transparent from the observation of Fig. 1(b), thus they had higher transmittance with comparing 150 nm samples at the same *R*[O_2_]% as shown in Fig. 5(a). Band gaps of 50 nm samples could be precisely confirmed for its crystal structure tend to be dominated only by AgO (002) peak, and widened from 0.8 to 1.7 eV with increasing *R*[O_2_]% from 10% to 17%. Meanwhile, from Fig. 4(c) and 5(c) the observation is that the resistivity (*ρ*) reached the summit value as *R*[O_2_]% = 20% of 150 nm Ag_*x*_O films: *ρ* = 3.2 × 10^6^ Ω·cm and as *R*[O_2_]% = 16% of 50 nm Ag_*x*_O films: *ρ* = 2.2 × 10^8^ Ω·cm. The resistivity of 50 nm samples is 2 magnitudes higher than that of 150 nm samples. The band gap reached the summit value as *R*[O_2_]% = 17% of 150 nm Ag_*x*_O films: *E*_G_ = 2.5 eV and 1.5 eV, and as *R*[O_2_]% = 17% of 50 nm Ag_*x*_O films: *E*_G_ = 1.7 eV. These results might be interpreted as that after *R*[O_2_]% exceeded 17% provided O ions sufficiency ambient in terms of the formation of the higher chemical state of Ag cations, which in the case of 150 nm depositions of Ag_*x*_O formed by sub-stabled Ag_3_O_4_ crystals.

**Fig. 6:**
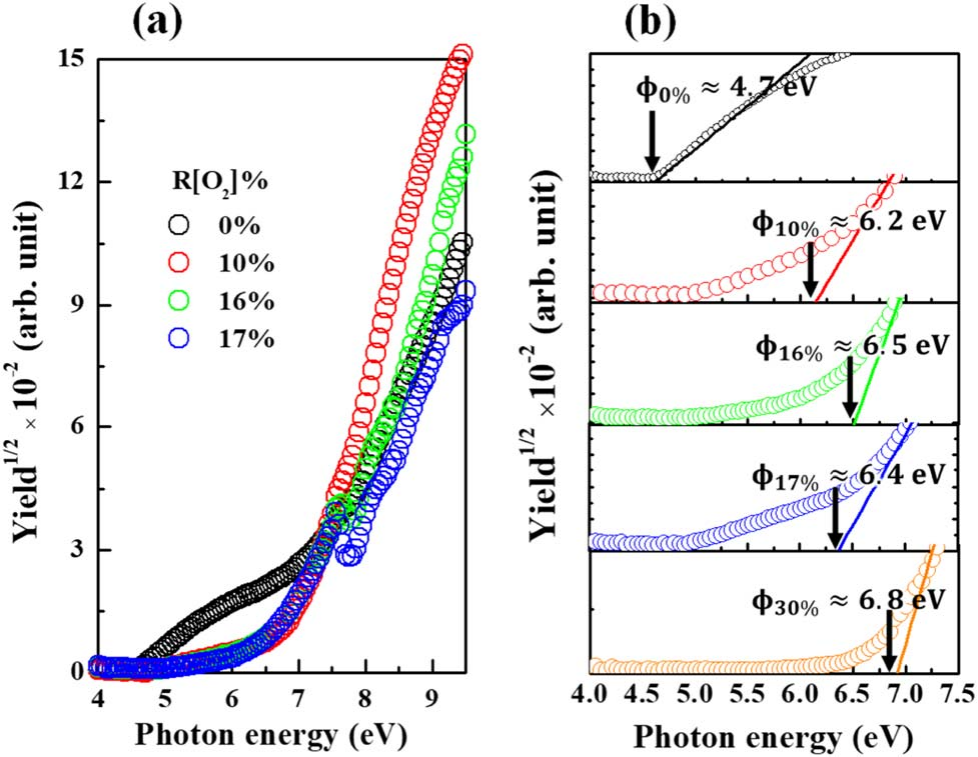
PYS spectra of Ag_*x*_O films with various *R*[O_2_]% (a) full range (b) magnification of small range with linear extrapolation to determine work function.

Afterward, all the measurements and analyses were based on the 150 nm thickness Ag_*x*_O sample. The variations in PYS spectra of Ag_*x*_O films depending on the *R*[O_2_]% are presented in Fig. 6 (a) full scan range and (b) magnification of small range for determining the work function of Ag and the ionization energy of Ag_*x*_O. The monochromatized photon from D_2_ (30 W) lamp is used as the excitation light. The density of yield photoelectrons (*Y*) of Ag_*x*_O samples is detected by irradiated D_2_ light with incremental photon energy (*hv*) scan from 4 eV to 9.5 eV, which is thought to be proportional to the square root in the surface area of *hv* over the threshold ionization energy (*I*_th_) which is equal to the energy difference *E*_0_ − *E*_F_ for Ag, or *E*_0_ − *E*_v_ for Ag_*x*_O, where *E*_0_ is vacuum level, *E*_F_ is Fermi level, *E*_v_ is valence band energy. *I*_th_ was evaluated through the following equation *Y* ∝ (*hv* − *I*_th_)^1/2^ (ref. 51 and 52) with plotting the *hv versus Y*^1/2^ spectra then by linear extrapolations of *Y*^1/2^.

In the certain range of increasing *R*[O_2_]%, the ionization energy of Ag_*x*_O tended to be high energy shifting of the spectra approaching *R*[O_2_]% = 10% obtained approximately 6.2 eV and by increasing *R*[O_2_]% to 16% shifted to 6.5 eV. However, when *R*[O_2_]% is 17%, the yield of photoelectrons was not monotonically increasing with increasing *hv* and the ionization energy seems to be shifted in the opposite direction reaching to 6.4 eV. This may be attributed to the highest resistivity of 17% Ag_*x*_O sample with mere electrons so that the 0.1 seconds waiting time yielded a lower density of photoelectrons, and *R*[O_2_]% = 16% sample had similar abnormal data. And after *R*[O_2_]% increased to 30%, its ionization energy continued shifting to high energy at 6.8 eV and there was no abnormal behavior of the yield of photoelectron as *R*[O_2_]% = 30%.

**Fig. 7:**
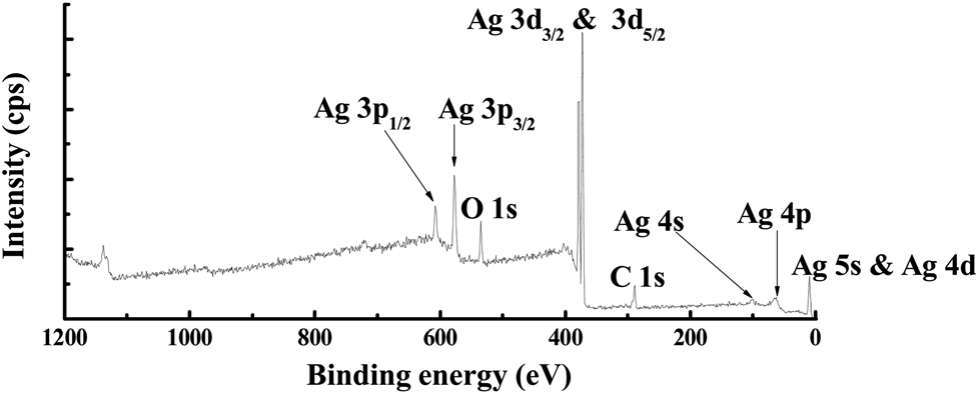
XPS survey spectrum obtained from the *R*[O_2_]% = 17% Ag_*x*_O sample.

**Fig. 8:**
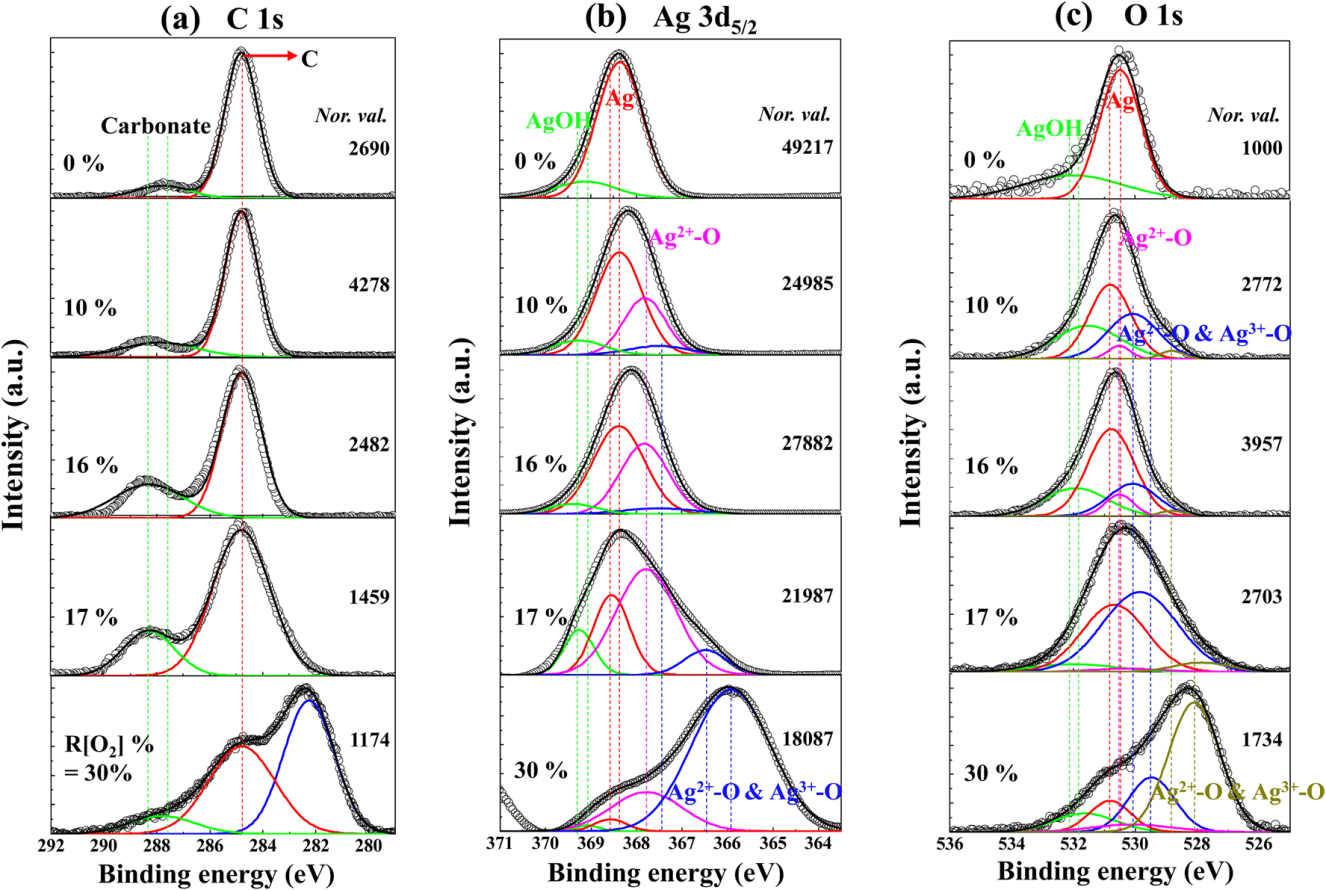
Measured HXPS data for (a) C 1s (b) Ag 3d_5/2_ (c) O 1s spectra of Ag_*x*_O films with various *R*[O_2_]%.

The XPS survey spectrum obtained from the *R*[O_2_]% = 17% of Ag_*x*_O film is shown in Fig. 7. The major elements and the minor elements represent peaks in this survey spectrum including the Ag 2p, Ag 3d_3/2_, Ag 3d_5/2_, Ag 4s, and Ag 4p peaks, the O 1s peak and the C 1s peak, and the Ag 5s & Ag 4d peak. The specific information of C 1s, Ag 3d_5/2_ and O 1s spectra about the peak assignments and chemical species correspond to different Ag–O compounds with the comparison of the increment of *R*[O_2_]% from 0% to 30% obtained by examining the high-resolution XPS spectra are shown in Fig. 8(a–c), respectively. And on the right side of Fig. 8 is the normalized value.

The binding energies are calibrated to the C 1s peak at 284.8 eV using charging correction and XPS analyzer techniques. Afterwards, the Ag 3d_5/2_, O 1s, and C 1s spectra were fitted with Gaussian curves. Referring to several previous published XPS studies for Ag_*x*_O,^53–58^ the binding energies of C 1s, Ag 3d_5/2_ and O 1s photoelectrons associated with different silver compounds and their chemical shifting values of various *R*[O_2_]% are given in Tables 2 and 3.

**Table 2:** Binding energies and chemical species of C 1s spectra

Peak assignment	C 1s
C-metal	282.2
C	284.8
Carbonate	288.4

**Table 3:** Binding energies and chemical shifts for different Ag_*x*_O compounds

Peak assignment	Ag 3d_5/2_	O 1s
Ag_3_O_4_	366.2	528.5
Ag_3_O_4_	367.5	530.2
AgO	367.7	530.5
Ag	368.4	530.5
AgOH	369.3	532

The C 1s spectra of Ag_*x*_O films with various *R*[O_2_]% are shown in Fig. 8(a). There are two distinct peaks shown in these spectra of the assignments at 284.8 eV because of adsorbed hydrocarbons and at 288.4 ± 0.02 eV attributed to the surface carbonate.^59–61^ With increments of the *R*[O_2_]% from 0% to 30%, the peak positions were shifted by 1 eV towards the high energy direction from 287.4 eV to 288.4 eV, in the peaks of surface carbonate their intensities also increased with increasing *R*[O_2_]%. From the GIXD data, we determined that with the increments of *R*[O_2_]%, the dominant compositions of Ag_*x*_O were presumably changed from Ag^2+^ to the mixture of randomly distributed Ag^2+^ and Ag^3+^. This may be caused by the surface Ag^3+^-adhered carbonate having higher binding energy than the surface Ag^2+^-adhered carbonate. However, for *R*[O_2_]% increment of 30%, the extraordinary peak appears as the third peak at 282.2 eV with significant intensity, which can be expected to be C–Ag bonds from previous studies.^62,63^

The Ag 3d_5/2_ and O 1s spectra of Ag_*x*_O with various *R*[O_2_]% are shown in Fig. 8(b and c). While the *R*[O_2_]% = 0%, the binding energies of Ag thin film can be observed at 368.4 eV of Ag peak in Ag 3d_5/2_ and at 530.5 ± 0.02 eV of Ag_*x*_O-related peak in the O 1s spectra. It is postulated that the Ag_*x*_O-related peak obtained from the Ag metal sample may be caused by the influence of the oxidized layer on the surface during atmospheric exposure. As evidence, the intensity of the O 1s peaks of the metallic Ag thin film is less than that of the corresponding peaks in Ag 3d_5/2_. And there are small peaks present at the higher binding energy side of both Ag 3d_5/2_ and O 1s spectra corresponding to 368.9 eV and 532 eV, respectively. This might indicate the presence of hydroxy groups of AgOH and they are most likely formed by the absorption of H_2_O during exposure to air. However, AgOH is not stable above 228 K,^53,57^ so it may be impossible for AgOH to exist in this case but here we only focus on the surface area. Meanwhile, as the *R*[O_2_]% increased to 10% and 16%, in the Ag 3d_5/2_ spectra of Ag peaks intensities were reduced and the proportional increments of Ag_*x*_O compound peaks appeared in comparison to *R*[O_2_]% = 0%. The binding energies of Ag 3d_5/2_ and O 1s were shifted to the lower binding energies within the increase in the oxidation state of Ag. Furthermore, we found that by intentionally increasing the *R*[O_2_]% from 16% to 17%, even increasing the *R*[O_2_]% by just one percent, the splitting peaks of the Ag_*x*_O compound shifted further to the lower binding energies. From the observation of the Ag 3d_5/2_ spectrum of *R*[O_2_]% = 17% deposition, AgO dominates the fitting fragments of the Ag oxidation state. Whereas in the increase of *R*[O_2_]% = 30%, the assignment of the peaks occurred further transitions toward lower binding energies at 366.2 eV of Ag 3d_5/2_ spectrum and 528.5 eV of O 1s spectrum, and incorporation of higher oxidation states formed as the mixture of AgO and Ag_2_O_3_. This suggests that at the higher oxygen flow ratio, the oxidation states of Ag are increased with the incorporation of more electrons into the chemical bonds^64^ to form the dominant oxidation states of Ag_3_O_4_, which is the mixture of two or more individual oxides of Ag^(II)^O and Ag_2_^(III)^O_3_.

**Fig. 9:**
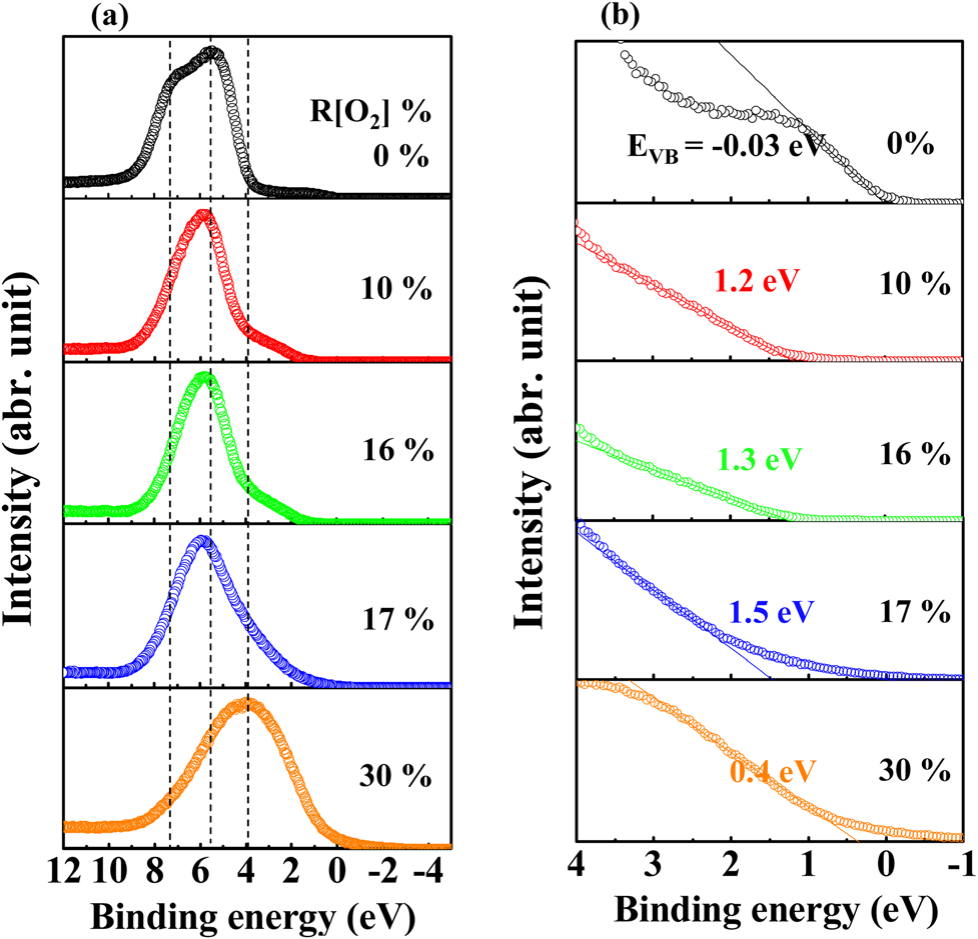
Measured XPS data for (a) the peaks of the valence-band region and (b) magnifying the valence-band region around *E*_F_ region of Ag_*x*_O films deposited at various *R*[O_2_]%.

The high resolution in the −1–10 eV scan of the valence band (VB) spectra obtained by XPS measurement of Ag_*x*_O films with various *R*[O_2_]% is shown in Fig. 9(a). The *R*[O_2_]% = 0% VB spectrum presents two peaks at the binding energy of 7.2 eV and 5.5 eV, which may be the result of the Ag 5s and 4d orbital atoms exerting repel forces on each other. This is consistent with the metal–metal interactions for the electric dipole transitions from 5s to 4d orbitals.^65,66^ Increasing the oxygen flow ratio of *R*[O_2_]% from 10% to 17% and 30%, only one peak can be observed with the binding energy changing from 5.8 eV to 4 eV. In the case of *R*[O_2_]% = 17% and 30%, the peak wing on the lower binding energy side was lifted to a higher intensity compared to *R*[O_2_]% = 10% and 16%. This may indicate that with increasing *R*[O_2_]%, the atomic interaction preference for metal (Ag) and metal (Ag) transition to metal (Ag) and nonmetal (O)^8^ is interpreted as the attractive forces between the charge of Ag 4d orbital and the charge of O 2p orbital bonding to form a hybrid orbital around the valence band.^18,61^ The feature of the lower binding energy shifting would be to the extent of hybridization of Ag 5s and/or 4p orbital with O 2p orbital.^67–69^ Meanwhile, Fig. 9(b) of magnifying the valence band region provides good evidence for the interaction of the metal (Ag) and nonmetal (O) bonding system. As *R*[O_2_]% = 0%, the density of states at the Fermi level can be properly observed. This is in agreement with the metallic behavior characterized as pure Ag. After supplying oxygen *R*[O_2_]%, we can observe the oxygen-induced band is forming. In addition to that, these results closely match the ionization energy or work function data from PYS measurements, which shows that high oxygen flow ratios tend to have more oxygen incorporated into the deposited film, especially the top surface range. Up to now, we can summarize all the information together to define the main species of Ag_*x*_O films; that is, the samples of Ag_*x*_O grown under the *R*[O_2_]% = 17% and 30% are the mixture of Ag^(II)^O and Ag_2_^(III)^O_3_ randomly distributed as Ag_3_O_4_, while those grown under *R*[O_2_]% = 10% and 16% are mainly Ag^(II)^O.

**Fig. 10:**
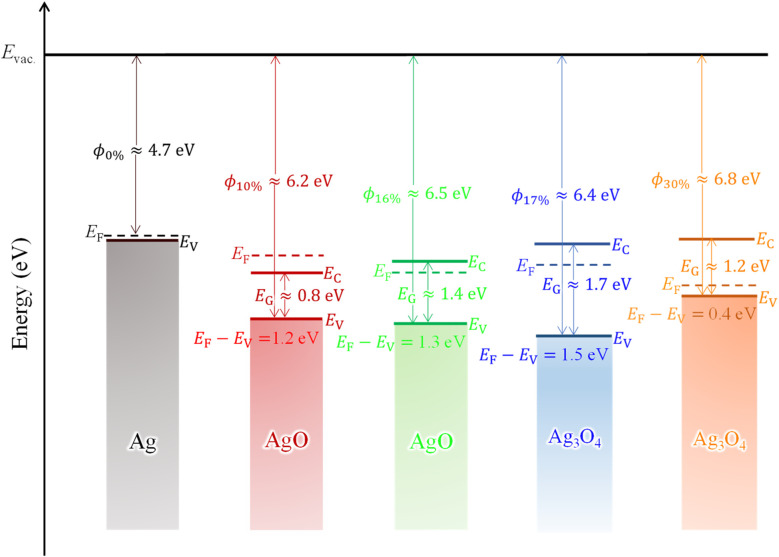
Schematic energy level diagram of Ag_*x*_O with various *R*[O_2_]%.

Lastly, combining all the causation and correlation, we established a pilot energy level diagram of Ag_*x*_O films with various *R*[O_2_]% to illustrate the mechanism between the material character and *R*[O_2_]% as shown in Fig. 10. And the comparisons of different *R*[O_2_]% influence on the physical parameters are summarized in Table 4. Note that the evaluation of the valence band level and the Fermi level difference from the vacuum level is employed using 150 nm Ag_*x*_O thin films, whereas the band gaps were employed using 50 nm Ag_*x*_O thin films because it was hard to obtain the data of band gaps using 150 nm Ag_*x*_O thin films. In polycrystalline materials, the changes in properties with thickness are generally not significant,^70,71^ and this is also confirmed by XRD, SEM, and transmittance measurements for the samples used in this study. For *R*[O_2_]% = 0%, the metallic Ag film was deposited with a conductivity of 10^6^ S·cm^−1^, work function of 4.7 eV, and valence band and conduction band overlapping at the 0 eV (measured data showed −0.03 eV and we thought this is due to air explosion) with respect to Fermi level. For *R*[O_2_]% = 10%, the semiconductor and/or insulator of AgO was deposited with the resistivity of 6.3 × 10^−5^ Ω·cm, the work function (*ϕ*) of 6.2 eV, and the band gap (*E*_G_) of 0.8 eV (estimated value), in which the bottom of the conduction band located at −0.4 eV and the top of the valence band located at −1.2 eV concerning the Fermi level. In general, the resistivity of the AgO thin film is around 1 Ω·cm.^72^ So, it can be seen that the conductivity of a transparent AgO film grown under *R*[O_2_]% = 10% is as high as that of a metal. However, from the transmittance results the characteristics of *R*[O_2_]% = 10% at the transparent region are different from those of other samples, and the reflection, not absorption, is dominant. In this case, it may not be possible to calculate the absorption edge well from the equation of Tauc.

**Table 4:** Comparison of the different *R*[O_2_]% Ag_*x*_O

*R*[O_2_]%	O-state	*ϕ* (eV)	*E*_G_ (eV)	*E*_V_ (eV)	|*E*_F_ − *E*_V_| (eV)
0%	Ag	4.7	0	−0.03	0.03
10%	AgO	6.2	0.8	1.2	1.2
16%	AgO	6.5	1.4	1.3	1.3
17%	Ag_3_O_4_	6.4	1.7	1.5	1.5
30%	Ag_3_O_4_	6.8	1.2	0.4	0.4

For *R*[O_2_]% = 16%, the semiconductor and/or insulator of AgO was deposited with a resistivity of 8.6 × 10^5^ Ω·cm, the work function (*ϕ*) of 6.5 eV, and the band gap (*E*_G_) of 1.4 eV. The bottom of the conduction band located at 0.1 eV and the top of the valence band located at −1.3 eV with respect to the Fermi level. For *R*[O_2_]% = 17% & 30%, the deposition sample is semiconductor and/or insulator of a mixture of Ag^(II)^O and Ag_2_^(III)^O_3_ randomly distributed as Ag_3_O_4_ with a resistivity of 4.1 × 10^5^ Ω·cm (*R*[O_2_]% = 17%) and 1.7 × 10^6^ Ω·cm (*R*[O_2_]% = 30%), the work function (*ϕ*) of 6.4 eV (*R*[O_2_]% = 17%) and 6.8 eV (*R*[O_2_]% = 30%), and the band gap (*E*_G_) that of 1.7 eV (*R*[O_2_]% = 17%) and 1.2 eV (*R*[O_2_]% = 30%). The bottom of the conduction band located at 0.2 eV (*R*[O_2_]% = 17%) and 0.8 eV (*R*[O_2_]% = 30%) and the top of the valence band located at −1.5 eV (*R*[O_2_]% = 17%) and −0.4 eV (*R*[O_2_]% = 30%) with respect to the Fermi level. However, the valence band energy level and the work function data of 16% and 17% may be unreliable because the high resistance leading to the photoelectron cannot be obtained clearly.

**Fig. 11:**
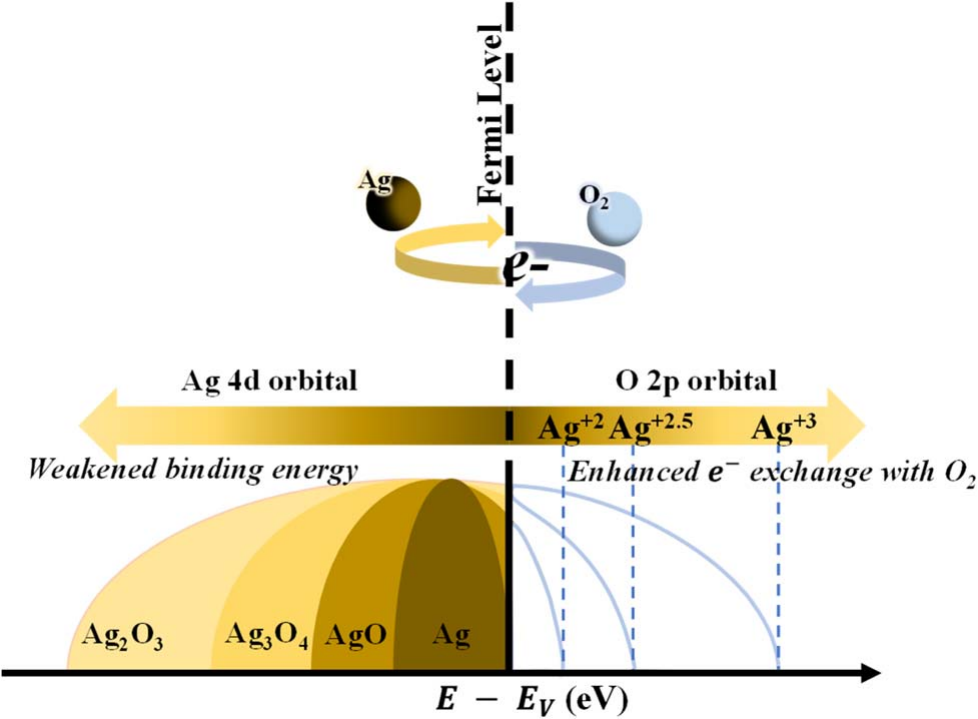
The transition mechanism of Ag_*x*_O with increasing *R*[O_2_]%.

The transition mechanisms between the metallic, semiconductor, and insulator states of Ag_*x*_O with increasing *R*[O_2_]% are shown in Fig. 11. When we checked the literature on silver oxide, we found that N. Qin *et al.* had investigated the valence band state in detail.^73^ According to this literature, the results obtained in this study can be understood more clearly. In short, a higher proportion of d-band holes and more electrons closer to the Fermi level under the higher valence Ag species were revealed in the literature from the computational studies, and our experimental results showed the same results.

## 4. Conclusions

The properties of Ag_*x*_O thin films with various *R*[O_2_]% have been investigated and analyzed using different types of measurements. GIXD spectra and SEM images were used for the crystal structure characterization, and UV-vis spectra and the resistance meter were used for the band gap and resistivity investigations. From GIXD data, it was found that Ag_*x*_O films tended to be a mixture of Ag^(II)^O and Ag_2_^(III)^O_3_. To precisely confirm the band gaps of Ag_*x*_O, two different thicknesses of 150 nm and 50 nm were deposited. The PYS spectra were used for work function confirmation. XPS was used to characterize the chemical states of Ag_*x*_O. With an increase in *R*[O_2_]% from 0% to 30%, Ag_*x*_O has a variational orientation for AgO (111) to Ag_3_O_4_ (002) grains, and the band gaps have extended to 1.7 eV but narrowed to 1.2 eV. The chemical states of Ag_*x*_O have been confirmed as AgO (*R*[O_2_]% = 10% and 16%) and Ag_3_O_4_ (*R*[O_2_]% = 17% and 30%). Therefore, Ag_*x*_O has the potential to be used as a transparent electrode in the infrared light range for heterojunctions and Schottky junctions. The energy level band has been given but the details of the physical phenomena need further confirmations.

## Data availability

The data supporting this article have been included as part of the ESI.†

## Conflicts of interest

There are no conflicts to declare.

## Supplementary Material

RA-014-D4RA02039A-s001

## References

[cit1] Yanagi H., Inoue S., Ueda K., Kawazoe H., Hosono H., Hamada N. (2000). J. Appl. Phys..

[cit2] Bock F. X., Christensenb T. M., Riversc S. B., Doucettea L. D., Lad R. J. (2004). Thin Solid Films.

[cit3] Rivers S. B., Bernhardt G., Wright M. W., Frankel D. J., Steeves M. M., Lad R. J. (2007). Thin Solid Films.

[cit4] Haynes W. M. , Lide D. R. and Bruno T. J., CRC Handbook of Chemistry and Physics, Internet Version 2005, Boca Raton, FL, 2005, http://www.hbcpnetbase.comCRCPress

[cit5] Linden D. and Reddy T. B., Handbook of Batteries, McGraw-Hill, New York, 3rd edn, 1995

[cit6] Smith D. F., Graybill G. R., Grubbs R. K., Gucinski J. A. (1997). J. Power Sources.

[cit7] Fortin E., Weichman F. L. (1964). Phys. Status Solidi.

[cit8] Kim J. B., Kim C. S., Kim Y. S. (2009). et al.. Appl. Phys. Lett..

[cit9] Minami T., Shimokawa K., Miyata T. (1998). J. Vac. Sci. Technol., A.

[cit10] Varkey A. J., Fort A. F. (1993). Sol. Energy Mater. Sol. Cells.

[cit11] Jie W., Lei Y., Jia H., Jia M., Hou H., Zheng Z. (2014). et al.. Dalton Trans..

[cit12] Wu Z. S., Yang S., Sun Y., Parvez K., Feng X., Müllen K. (2012). 3D nitrogen-doped graphene aerogel-supported Fe_3_O_4_ nanoparticles as efficient electrocatalysts for the oxygen reduction reaction. J. Am. Chem. Soc..

[cit13] Luo Y.-R. , Handbook of Bond Dissociation Energies in Organic Compounds, CRC Press, 1st edn, 2002, 10.1201/9781420039863

[cit14] Holewinski A., Idrobo J. C., Linic S. (2014). High-performance Ag-Co alloy catalysts for electrochemical oxygen reduction. Nat. Chem..

[cit15] Xiao J., Wan L., Wang X., Kuang Q., Dong S., Xiao F., Wang S. (2014). Mesoporous Mn_3_O_4_–CoO Core–Shell Spheres Wrapped by Carbon Nanotubes: A High Performance Catalyst for the Oxygen Reduction Reaction and CO Oxidation. J. Mater. Chem. A.

[cit16] Yin J., Li Y., Lv F., Fan Q., Zhao Y. Q., Zhang Q., Wang W., Cheng F., Xi P., Guo S. J. (2017). NiO/CoN Porous Nanowires as Efficient Bifunctional Catalysts for Zn–Air Batteries. ACS Nano.

[cit17] Cao Y., Wei Z., He J., Zang J., Zhang Q., Zheng M., Dong Q. (2012). α-MnO_2_ Nanorods Grown *in situ* on Graphene as Catalysts for Li–O_2_ Batteries with Excellent Electrochemical Performance. Energy Environ. Sci..

[cit18] Asbalter J., Subrahmanyam A. (2000). J. Vac. Sci. Technol., A.

[cit19] Ishida T., Kobayashi H., Nakato Y. (1993). J. Appl. Phys..

[cit20] Boronin A. I., Koscheev S. V., Murzakhmetov K. T., Avdeev V. I., Zhidomirov G. M. (2000). Appl. Surf. Sci..

[cit21] Gao X. Y., Wang S. Y., Li J., Zheng Y. X., Zhang R. J., Zhou P., Yang Y. M., Chen L. Y. (2004). Thin Solid Films.

[cit22] Pettersson L. A. A., Snyder P. G. (1995). Thin Solid Films.

[cit23] Subrahmanyam A., Barik U. K. (2007). Indium doped silver oxide thin films prepared by reactive electron beam evaporation technique: electrical properties. J. Mater. Sci..

[cit24] Barik U. K. (2003). *et al.*, Electrical and optical properties of reactive DC magnetron sputtered silver oxide thin films: role of oxygen. Thin Solid Films.

[cit25] Allen M., Durbin S. (2008). Appl. Phys. Lett..

[cit26] Dang G. T., Kawaharamura T. (2015). et al.. Appl. Phys. Lett..

[cit27] Magari Y., Furuta M. (2020). et al.. Appl. Surf. Sci..

[cit28] Liu X., Dang G. T., Liu L., Kawaharamura T. (2022). Appl. Surf. Sci..

[cit29] Van Ingen R. P., Fastenau R. H. J., Mittemeijer E. J. (1994). J. Appl. Phys..

[cit30] Werner A., Hochheimer H. D. (1982). Phys. Rev. B: Condens. Matter.

[cit31] Stehlik B., Weidenthaler P., Vlach J. (1959). Collect. Czech. Chem. Commun..

[cit32] Standke B., Jansen M. (1987). J. Solid State Chem..

[cit33] Brese N. E., O'Keeffe M., Ramakrishna B. L., Von Dreele R. B. (1990). J. Solid State Chem..

[cit34] Waterhouse G. I. N., Bowmaker G. A., Metson J. B. (2001). Oxidation of a polycrystalline silver foil by reaction with ozone. Appl. Surf. Sci..

[cit35] Barteau M. A., Madix R. J. (1983). J. Electron Spectrosc. Relat. Phenom..

[cit36] Hoflund G. B., Hazos Z. F., Salaita G. N. (2000). Phys. Rev. B: Condens. Matter Mater. Phys..

[cit37] Bock F., Christensen T., Rivers S. (2004). et al.. Thin Solid Films.

[cit38] Pierson J. F., Rousselot T. C. (2005). Surf. Coat. Technol..

[cit39] Tseng C. C. (2010). *et al.*, Effects of deposition and annealing temperatures on the electrical and optical properties of Ag_2_O and Cu_2_O–Ag_2_O thin films. J. Vac. Sci. Technol., A.

[cit40] Schmidt A. A., Offermann J., Anton R. (1996). The role of neutral oxygen radicals in the oxidation of Ag films. Thin Solid Films.

[cit41] Fortin E., Weichman F. L. (1964). photoconductivity in Ag_2_O. Phys. Status Solidi B.

[cit42] Suzuki R. O., Ogawa T., Katsutoshi O. (1999). Use of ozone to prepare silver oxides. J. Am. Ceram. Soc..

[cit43] Usategui M. , Higher Oxidation States of Silver, LSU Historical Dissertations and Theses, Louisiana State University and Agricultural & Mechanical College, 1961, vol. 658, 10.31390/gradschool_disstheses.658

[cit44] Burkhard S., Jansen M. (1987). J. Solid State Chem..

[cit45] Fischer P., Jansen M. (1990). Cyclovoltammetrische-und Röntgenbeugungsuntersuchungen zur anodischen abscheidung Höherer silberoxide. Solid State Ionics.

[cit46] Mansour A. N. (1990). Evidence for an Ag_4_O_3_ phase of silver oxide. J. Phys. Chem..

[cit47] McMillan J. A. (1960). Magnetic properties and crystalline structure of AgO. J. Inorg. Nucl. Chem..

[cit48] Graff W. S., Stadelmaier H. H. (1958). Higher oxides of silver. J. Electrochem. Soc..

[cit49] Hamberg I., Granqvist C. G. (1986). Evaporated Sn-doped In_2_O_3_ films: Basic optical properties and applications to energy-efficient windows. J. Appl. Phys..

[cit50] Urbach F. (1953). The long-wavelength edge of photographic sensitivity and of the electronic absorption of solids. J. Electrochem. Soc..

[cit51] Nakayam Y., Ishii H. (2008). et al.. Appl. Phys. Lett..

[cit52] Hisao I. , Photoelectron yield spectroscopy, Compendium of Surface and Interface Analysis, 2018, pp. 457–463, 10.1007/978-981-10-6156-1_75

[cit53] Li W.-X., Stampfl C., Scheffler M. (2003). Insights into the function of silver as an oxidation catalyst by *ab initio* atomistic thermodynamics. Phys. Rev. B: Condens. Matter Mater. Phys..

[cit54] Weaver J. F., Hoflund G. B. (1994). Surface characterization study of the thermal decomposition of Ag_2_O. Chem. Mater..

[cit55] Gaarenstroom S. W., Winograd N. J. T. J. (1977). Initial and final state effects in the ESCA spectra of cadmium and silver oxides. J. Chem. Phys..

[cit56] Rehren C. (1991). *et al.*, Surface and subsurface products of the interaction of O_2_ with Ag under catalytic conditions. Catal. Lett..

[cit57] Schön G. (1973). *et al.*, ESCA studies of Ag, Ag_2_O and AgO. Acta Chem. Scand..

[cit58] Hammond J. S., Gaarenstroom S. W., Winograd N. (1975). X-ray photoelectron spectroscopic studies of cadmium-and silver-oxygen surfaces. Anal. Chem..

[cit59] Savitzky A., Golay M. J. E. (1964). Anal. Chem..

[cit60] Bukhtiyarov V. I. (1989). *et al.*, The state of oxygen on the surface of polycrystalline silver. React. Kinet. Catal. Lett..

[cit61] Charles T. C., Mark T. (1984). Paffett. Surf. Sci..

[cit62] Zhang G., Yan P., Wang P., Chen Y., Zhang J. (2007). The preparation and mechanical properties of Al-containing a-C: H thin films. J. Phys. D Appl. Phys..

[cit63] Baba K., Hatada R. (2003). Deposition and characterization of Ti- and W-containing diamond-like carbon films by plasma source ion implantation. Surf. Coat. Technol..

[cit64] Behrens P. (1992). Bonding in silver-oxygen compounds from Ag L_3_ XANES spectroscopy. Solid State Commun..

[cit65] Bigelow R. W. (1988). An XPS study of air corona discharge-induced corrosion products at Cu, Ag and Au ground planes. Appl. Surf. Sci..

[cit66] Jansen M. (1987). Homoatomic d10–d10 interactions: their effects on structure and chemical and physical properties. Angew Chem. Int. Ed. Engl..

[cit67] Behrens P. (1999). Z. Anorg. Allg. Chem..

[cit68] Romand M., Roubin M., Deloume J. P. (1978). J. Electron Spectrosc. Relat. Phenom..

[cit69] Tjeng L. H., Meinders M. B. J., van Elp J., Ghijsen J., Savatsky G. A., Johnson R. L. (1990). Phys. Rev. B.

[cit70] Fan H., Li Z., Huang M., Zhang X. (2011). Thickness effects in polycrystalline thin films: Surface constraint *versus* interior constraint. Int. J. Solids Struct..

[cit71] Alzaid M., Mohamed W. S., El-Hagary M., Shaaban E. R., Hadia N. M. A. (2021). Opt. Mater..

[cit72] Tvarusko A. (1968). J. Electrochem. Soc..

[cit73] Qin N., Yu S., Ji Z., Wang Y., Li Y., Gu S., Gan Q., Wang Z., Li Z., Luo G., Zhang K., Lu Z. (2022). CCS Chem..

