# Supplementary material for: The effects of oxygen flow ratio on the properties of AgxO thin films grown by radio frequency magnetron sputtering
Source: RSC Adv. 2024 Jul 23;14(32):23215–24. doi: 10.1039/d4ra02039a (PMC11264336; doi:10.1039/d4ra02039a)
Supplement: RA-014-D4RA02039A-s001 [file RA-014-D4RA02039A-s001.pdf]

Electronic Supplementary Material (ESI) for RSC Advances.
This journal is © The Royal Society of Chemistry 2024

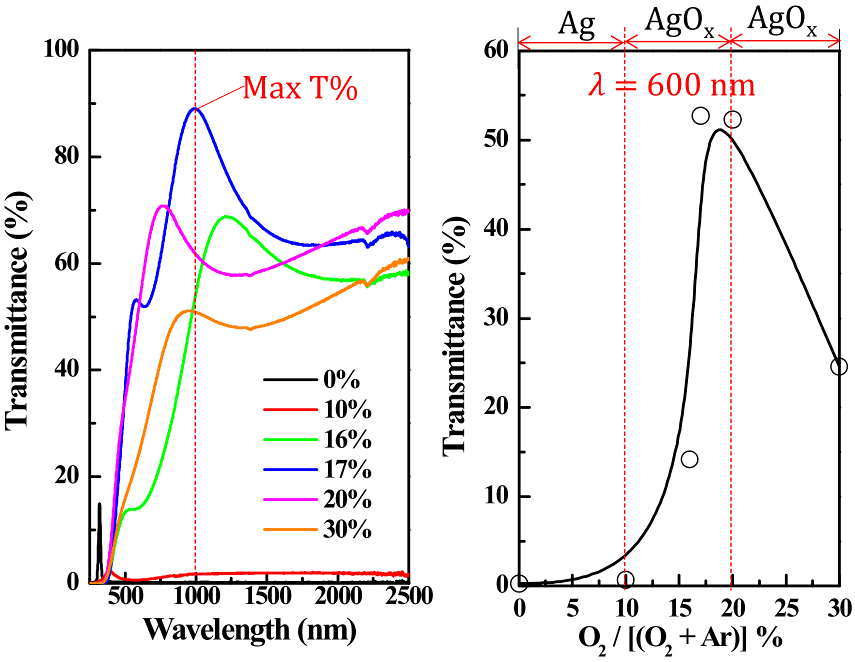

Fig. 1 Optical transmittance and the transmittance of 150 nm samples at a wavelength of 600nm are plotted as a function of O_2 flow ratio.

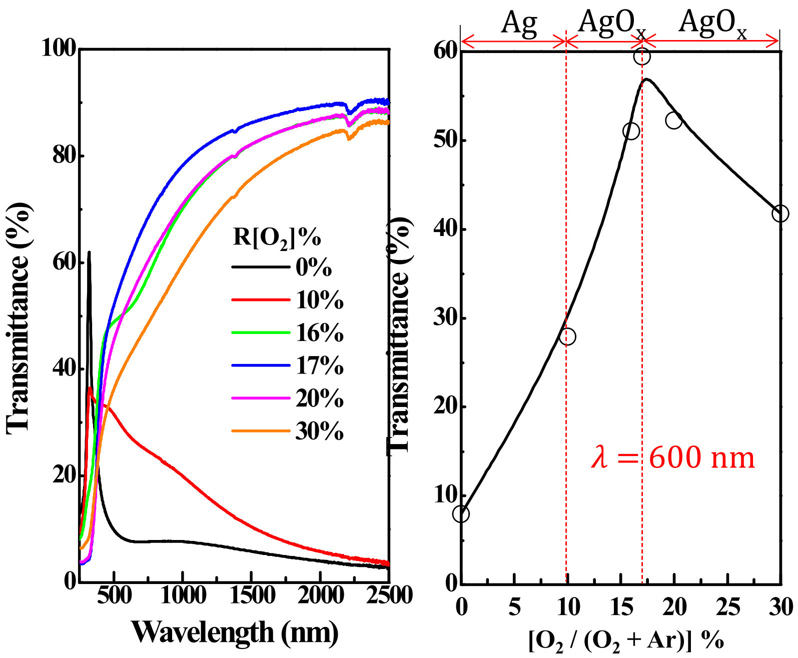

Fig.2 Optical transmittance and the transmittance of 50 nm samples at a wavelength of 600nm are plotted as a function of O_2 flow ratio.
